# OMEGA-3 IN ARABIAN GULF FISH

**Published:** 2010

**Authors:** H.A. Hajar Albinali

**Affiliations:** * *Chairman, Department of Cardiology and Cardiothoracic Surgery, Hamad Medical Corporation, Doha, Qatar; Adviser to H.H. the Emir for Health Affairs, Founder and President of Gulf Heart Association (GHA); Minister of Public Health, Qatar (1999-2005); Undersecretary of Health, Qatar (1981-1993)*

## PART VI

Final article in a 6-part series on the cardiac health benefits of omega-3PUFA and omega-3 content in Arabian Gulf fish.

In my first article in this series I said: “I will be starting one page devoted to fish in each issue of Heart Views, discussing one fish type at a time with photographic illustration of the fish^1^.” Later on I broke that promise and instead of one page article, I got carried on to write several pages.

I focused on the cardiac beneficial effects of omega-3 poly unsaturated fatty acids (PUFA) in primary prevention, ischemic heart disease and myocardial infarction, heart failure, atherosclerosis, and atrial fibrillation. I did not extend my discussion to its other health benefits outside the heart such as stroke. Higher levels of fish (or omega-3 fatty acid) intake was associated with lower risk of total stroke, especially thrombotic and lacunar infarction. Potential mechanisms between the stroke/fish relationship include inhibition of platelet aggregation, lowered blood viscosity, suppressed formation of leukotrienes (mediators of neutrophil and macrophage aggregation), and reductions in plasma fibrinogen, blood pressure, and insulin resistance^2^.

In this article I want to conclude by listing the Arabian Gulf fish with high or low omega-3 poly unsaturated fatty acids (PUFA) content in the two tables bellow. The local Arabic name of the fish and the corresponding common English name are provided in the tables.

**Table 1:** Gulf Fish with high and medium ω-3 PUFA content

Common local name	Common English name	Fat content of edible portion (%)
Ooma	Sardines	13.00
Yanam	Grey Sweet Lip	3.90
Beyah	Mullet	1.80
Karari	Crevalle	1.70
Zobaidy	Malabar Cavalla	1.60
Kanaad	King Mackerel	1.60
Naisar	Black Spot Snapper	1.50
Jash	Orange Spot Trevally	1.50
Garfa	Black-Fin Crevalle	1.30
Badah	Common Mojarra	1.10

**Table 2:** Gulf Fish with low level of ω-3 PUFA content

Common local name	Common English name	Fat content of edible portion (%)
Gubgub	Sea Crab	1.00
Saffi	Rabbit Fish	0.80
Sikin	Cobia	0.70
Hamrah	Red Snapper	0.70
Rabeeb	Golden Trevally	0.66
Rubian	Shrimp	0.50
Faskar	Porgy	0.45
Jarjour	Grey Dog Shark	0.40
Hamour	Greasy Grouper	0.35
Sheary	Orange Emperor	0.27

The Qatar Ministry of Public Health (MPH) laboratory has determined that Sardines had the highest content of omega-3 PUFA followed by Grey Sweet Lip (Yanam). Medium levels were found in Malabar Cavalla, Orange Spot Travally, Crevalle, Black Spot Snapper, Black-Fin Crevalle, King Mackerel and Mullet^3^.

Low levels were found in Common Mojarra, Golden Trevally, Red Snapper, Cobia, Rabbit Fish, Porgy, Greasy Grouper, Grty Dog Shark and Orange Emperor. Low levels were also found in crab and shrimp3.

The Qatar Ministry of Public Health laboratory study showed that there are several species of Arabian Gulf fish considered as good sources of the omega-3 polyunsaturated fatty acids. The widely popular species of Greasy Grouper was found, however, a-poor-source for these essential fatty acids as I had discussed in a previous article^4^.

Finally, I would like to end up these this series of articles about fish in the Arabian Gulf to one peculiar fish that I had special personal experience with. I will discuss this fish not as food for consumption but as intellectual amusement and entertainment.

## The Parrotfish

Parrotfish is a very colorful fish that has many unique characteristics. It is a fascinating fish to see in its natural environment in the sea. The flesh of this fish was not analyzed by MPH laboratory because it is rarely consumed by Gulf citizens. In the Gulf, we never fish for Parrotfish because we do not eat it but it gets caught occasionally in fishing cages. Why a Parrotfish would enter those cages is mystery to me because it does not eat what other fish eats. My guess is that it probably enter for the algae-covered stone in the cage (you will see why bellow). I have never tasted Parrotfish, but some foreigners in Qatar consider it delicacy. In Polynesia, it is served raw and was once considered “royal food,” only eaten by the king^5^.

Perhaps no other fish is as unique as the Parrotfish. It is not only colorful but has amazing ability for changing colors. Parrotfish is not named for its brilliant colors, but because of its beak-like mouth. It has fused teeth that resemble a parrot’s beak and hence the name, “parrot fish”. Their powerful cutting-edged beaks are formed of fused incisor, like jaw teeth. With these they scrape from the surface of coral, algae, polyps, and other small plant and animal life upon which they feed.

Parrotfish is very unusual animal, because it also has a set of grinding teeth, located in the throat, in front of the esophagus. The latter teeth further break up the food to prepare it for the action of digestive enzymes^6^.

Parrotfish is mostly a vegetarian fish but the final products of its digestion, its feces, are sand. I like to call this fish a *sand factory*. This is how it produces it sand. As the algae are removed, bits of coral are removed with it and passed to the throat where pharyngeal teeth supply a kind of chewing surface. So with this parrot-like beak this fish grasps algae from coral and other rocky substrates. The pharyngeal teeth grind up coral rock that the fish ingests during feeding. After it digests, it excretes the rock as sand helping to create small islands on the sandy beaches. One Parrotfish can produce 90kg of sand each year^7^.

The development of Parrotfish is complex and accompanied by a series of changes in color. The sex life of Parrotfish is like that of Hamour (grouper) fish^4^, starting as female and then changing to male as it grows older. However, in many species a number of individuals develop directly to males (i.e. they do not start as females)^8^.

Parrotfishes are secretive, spending a great deal of time hidden away. This is largely due to their instincts of survival and also due to the fact that they eat things that are tough to get to sometimes. They have a unique way of eating with their hardened and modified mouths.

One of the more interesting characteristics of the Parrotfish is their preference to sleep during the night at the bottom of the reef. Some species protect themselves by burrowing into the sandy bottom where they remain until morning. Other species of Parrotfish excrete a mucus cocoon, particularly at night. Prior to going to sleep they cover themselves with a type of slimy covering forming a kind of cocoon in which to sleep, which protects them from nocturnal predators. This mucus envelope hides its scent from predators and may also act as an early warning system, allowing the parrotfish to flee when it detects predators disturbing the protective membrane. The mucus has antioxidant properties that may serve to repair bodily damage, or repel parasites, and to provide added protection from UV light. When morning arrives the fish discards the cocoon and goes about its business^9^.

## The Parrotfish and me

In 1993, I went fishing in a large yacht with friends near a Qatari Island called Halool, 80km north east of the city of Doha. I went down in a small boat for free diving adventure near the shore, carrying a fishing arrow with me. It ended in an unintentional killing of a beautiful Parrotfish. It is called “Gain” in local Arabic. I felt sorry for hitting that fish. I carried it back to the yacht for photography. While I was looking at it I wrote a poem about it. I cannot translate it to English as poetry, but I will try to translate the meaning.

In Halool sea, I went diving alone,

Next to a mighty large stone.

I was so amused to see,

Many beautiful creatures under the sea.

It was like a sea garden, a paradise in a heavenly place,

For sea creatures, not for human race.

Near a rock, a beautiful “Gain” could be seen,

Wearing a colorful dress, like a young queen,

Heading toward my arrow, swimming with grace,

She was like a beautiful creature from outer space.

In greens she was dressed,

And jewelry shone on her chest;

Her eye was like clear pearl, without shade,

Surrounded with green and red jade.

With movement of her tail she dances,

Like a young lady with romances.

Suddenly my arrow took off in a burst,

And landed in her green chest.

I cried with sorrow that took my breath,

For her accidental cruel death.

I never wanted to inflict pain,

On that beautiful Gain.

Because my heart is soft and tender,

I was never fit to be a hunter.

My deep and strong sorrow,

Forced me to break the arrow.

From such fishing, I decided to abstain,

I never went fishing in that place again.
